# Supplementary material for: Global hypo-methylation in a proportion of glioblastoma enriched for an astrocytic signature is associated with increased invasion and altered immune landscape
Source: eLife. 2022 Nov 22;11:e77335. doi: 10.7554/eLife.77335 (PMC9681209; doi:10.7554/eLife.77335)
Supplement: Figure 2—source data 1. [file elife-77335-fig2-data1.zip › Figure_2_source_data_1/Figure_2B/homerResults/motif50.similar.html]

motif50

## Information for motif50

C
T
A
G
A
G
T
C
C
T
A
G
A
T
C
G
A
G
C
T
G
A
T
C
G
A
C
T
A
C
T
G
A
C
T
G
A
G
C
T
A
G
T
C
G
C
T
A
C
A
G
T
A
T
C
G
G
A
T
C
A
G
C
T
A
G
C
T
C
A
G
T
T
C
A
G
A
G
T
C
G
T
A
C
G
T
C
A
A
T
G
C
G
C
A
T
A
T
C
G
A
C
G
T
C
T
A
G
T
A
G
C
G
A
C
T
A
G
T
C
G
C
T
A
A
C
T
G
T
G
A
C
C
T
G
A
A
T
C
G
A
G
C
T
A
C
T
G
G
C
T
A
C
A
T
G
T
A
C
G
A
C
T
G
G
C
A
T
G
T
A
C
C
A
G
T
A
G
C
T
  
Reverse Opposite:  

T
C
G
A
G
T
C
A
C
A
T
G
C
G
T
A
T
G
A
C
A
T
G
C
G
T
A
C
C
G
A
T
T
G
A
C
T
C
G
A
T
A
G
C
G
A
C
T
A
C
T
G
T
G
A
C
C
G
A
T
T
C
A
G
C
T
G
A
A
T
C
G
G
A
T
C
T
G
C
A
T
A
G
C
C
G
T
A
A
T
C
G
C
A
G
T
A
C
T
G
T
C
A
G
A
G
T
C
G
T
C
A
T
C
G
A
T
C
G
A
C
T
A
G
T
A
G
C
G
T
C
A
C
G
A
T
T
C
A
G
T
C
G
A
T
G
A
C
T
A
G
C
C
G
T
A
C
T
A
G
C
T
G
A
T
A
G
C
G
A
T
C
T
C
A
G
G
A
T
C
  

|  |  |
| --- | --- |
| p-value: | 1e-29 |
| log p-value: | -6.743e+01 |
| Information Content per bp: | 1.736 |
| Number of Target Sequences with motif | 72.0 |
| Percentage of Target Sequences with motif | 3.12% |
| Number of Background Sequences with motif | 2.4 |
| Percentage of Background Sequences with motif | 0.69% |
| Average Position of motif in Targets | 101.8 +/- 48.3bp |
| Average Position of motif in Background | 129.1 +/- 11.5bp |
| Strand Bias (log2 ratio + to - strand density) | -0.2 |
| Multiplicity (# of sites on avg that occur together) | 1.04 |
| Motif File: | file (matrix) reverse opposite |

### Similar de novo motifs found

|  |  |  |  |  |  |  |  |
| --- | --- | --- | --- | --- | --- | --- | --- |
| Rank | Match Score | Redundant Motif | P-value | log P-value | % of Targets | % of Background | Motif file |
| 1 | 0.812 | C G A T A T C G G A T C C A T G A T C G A G C T A T G C C G A T A C T G A C T G G A C T A T G C C G T A A C T G C A T G G C A T G A C T A G C T G C A T C A T G G A T C A G T C G C T A A G T C G C A T C A T G C G A T A C T G G A T C G A T C G A T C C G T A A C T G G T A C C G T A C A T G C G A T C T A G C T G A A C T G | 1e-21 | -48.580752 | 3.25% | 1.08% | motif file (matrix) |
| 2 | 0.796 | C A G T A T C G T A G C C A T G A T C G A G C T A T G C C G A T A C T G A T C G A C G T A T G C G C T A C A T G A T C G A G C T A G C T A G C T A C G T A T C G A T G C G T A C G T C A A T G C G C A T A T C G C A G T A T C G A T G C G A T C G T A C C G T A A T C G T A G C G T C A | 1e-14 | -34.312732 | 2.77% | 1.00% | motif file (matrix) |
| 3 | 0.835 | G C A T C A T G A G C T C A T G A T G C A G C T G A T C C G A T C A T G A C G T A C G T A T G C G C A T A C T G A T C G G A C T G A C T A G C T C G A T A C T G G A T C G A T C G C T A A T G C C G A T A T C G G C A T T C A G G A T C G A C T G A T C C G T A A C G T A T G C G C T A C A T G C A G T A T C G G C A T A C T G A T C G C A T G C G A T G A T C A G C T A G C T A G C T A C G T A T C G A G T C | 1e-7 | -17.917348 | 5.32% | 3.28% | motif file (matrix) |
